# Editorial: Gesture-Speech Integration: Combining Gesture and Speech to Create Understanding

**DOI:** 10.3389/fpsyg.2021.732357

**Published:** 2021-07-23

**Authors:** Naomi Sweller, Kazuki Sekine, Autumn B. Hostetter

**Affiliations:** ^1^Department of Psychology, Faculty of Medicine, Health and Human Sciences, Macquarie University, Sydney, NSW, Australia; ^2^Department of Human Informatics and Cognitive Science, Faculty of Human Sciences, Waseda University, Saitama, Japan; ^3^Department of Psychology, Kalamazoo College, Kalamazoo, MI, United States

**Keywords:** gesture, semantic meaning, comprehension, communication, cognitive load, individual differences, language

Gestures and speech are tightly linked. Since McNeill (1992) argued that gesture and speech form a single integrated system, research has shown that gestures and speech interact with each other across a variety of domains. Listeners can benefit from observing a speaker's gestures (e.g., Kelly, 2001), and similarly, speakers demonstrate improved communication and task performance when they gesture (e.g., Cook et al., 2010). In 15 articles, this Special Issue further examines how gesture and speech are integrated during speaking and listening. The functions of gesture, and potential mechanisms underlying gesture's beneficial effects are considered, and together, these articles highlight the impact that both producing and observing gestures can have on individuals' learning and communication across the lifespan. Here, we summarize some of the overarching themes that emerge from this collection.

Gesture seems to activate semantic meanings that are useful for comprehension and learning. Hughes-Berheim et al. found that participants' ratings of the semantic congruency of gesture-word pairs were similar, regardless of whether the word was presented in speech or in text. This suggests that gestures activate semantic meanings that are independent of language modality. Further, the meanings conveyed by gesture are particularly helpful for children's learning. Guarino and Wakefield examined 4–11-year-old children's understanding of instructions presented through speech alone, or through a combination of speech and gesture. They found a benefit of the combination of gesture and speech beyond speech alone that was most marked for 5-year-old children. Eye-tracking results suggested that the gestures may have helped children to organize their attention and clarify ambiguous spoken instructions. In addition to these attention-related functions, the semantic meaning activated by gesture can act as a cue during retrieval to help children remember what they learned. Mertens and Rohlfing compared progressively reduced iconic gestures with fully executed iconic gestures during children's recall of words. Although children's recall of the target words was unaffected by the type of gesture observed, their production of the target words at test was enhanced by progressively reduced gestures relative to fully executed gestures.

By activating semantic meaning, gestures help speakers and listeners resolve ambiguous references. Debreslioska and Gullberg examined the relationship between the information status of a referent (brand-new vs. inferable referants) and gesture, finding that gestures were more frequent with inferable than with brand-new referents. This finding suggests a function of gestures for disambiguating discourse content. Hinnell and Parrill found that listeners relied on a speaker's gesture as an indication of what the speaker's own opinion was. Speakers presented two contrasting ideas, and then said which one they agreed with. When the speaker accompanied their spoken agreement with a gesture, listeners were more likely to state that the speaker preferred the idea accompanied by the gesture. In this way, gestures activate semantic meaning that helps listeners infer what is meant when speech is not completely clear.

Even without accompanying speech, the semantic meanings conveyed by gesture are important for communication. Marentette et al. showed this in children's production of pantomime gestures, or non-co-speech gestures, that were performed during children's spoken narratives. Marentette et al. found that narratives that included non-co-speech gestures were longer and sometimes of higher quality than those with only co-speech gestures, suggesting that expressing information uniquely in gesture (and not in speech) can improve the overall quality of children's narratives. Hsu et al. make a similar point based on their analysis of gestures taken from a corpus of American TV talk shows. They discuss many examples of what they call “speech-embedded nonverbal depictions,” that is, non-verbal communicative cues presented iconically, but without simultaneously co-occurring speech. The authors argue that such depictions are frequently overlooked in the literature, and argue for their theoretical and functional significance. Taken together, these papers demonstrate how gestures activate semantic meanings that do not rely on accompanying speech and that contribute to the on-going narrative.

The benefits of gesture for comprehension also go beyond the purely semantic; gestures can also affect other areas of language processing from low-level phonemic recognition to high-level social judgments about the speaker. Hoetjes and Maastricht examined second language (L2) phoneme acquisition, with a focus on the complexity of both the phonemes and of the gestures observed. Gestures were either simple (pointing) or more complex (iconic) gestures, and the to-be-learned Spanish phonemes were either simple (contained in the Dutch phoneme inventory) or complex (not contained in the Dutch phoneme inventory). While the more complex gesture enhanced learning of the simple phoneme, it was detrimental to learning the complex phoneme. At the other end of the spectrum, gestures can also affect high-level social judgments about a speaker. Billot-Vasquez et al. found that native Mandarin and Japanese speakers evaluated the accents of non-native speakers and the non-native speakers themselves more favorably when they produced a familiar emblematic gesture with their speech compared to producing the speech alone. These papers suggest that gestures can contribute more to language than just activating a particular semantic meaning.

Even as gestures have these positive effects, they may also come with costs in some situations. Specifically, producing or processing a gesture may impose an additional cognitive cost for some speakers and listeners. This was shown by Rohrer et al. in the case of beat gestures (rhythmic hand movements without any semantic meaning) that accompanied speech in a listener's non-native language. Specifically, French intermediate learners of English watched a video of a speaker describing a short narrative event in either French or English using either beat gestures or no gesture. When the learners drew a depiction of the narrative, it was found that recall of the narrative was negatively affected by beat gestures when the narrative was presented in their non-native language. The authors propose that these gestures may have increased cognitive load. Further, Overoye and Wilson examined gesture's effects on working memory load during a verbal reasoning task. Gesturing while explaining verbal analogies did not alleviate the load on working memory (as has been shown in previous studies—e.g., Goldin-Meadow et al., 2001), but rather led to poorer performance on a secondary task than being prohibited from gesturing.

How can gestures simultaneously be helpful in some ways and detrimental in others? One possibility is that it depends on the speakers' or listeners' cognitive skillset. Such is the suggestion in Özer and Göksun's timely review article, in which they explore how individual differences in cognitive capacity might affect people's gesture production, and the extent to which they employ gesture as a tool for comprehension. Özer and Göksun conclude that gestures can be used as a tool to compensate for a lack of cognitive resources, by both speakers and listeners. Indeed, it is well-recognized that speakers' gestures are affected by individual differences, including cognitive skills and also neurodevelopmental factors. For example, Huang et al. discuss how the gestures produced by Chinese-speaking children on the autism spectrum differ from those of their typically developing peers.

The potential for gesture production to differ depending on a speaker's cognitive skillset is further explored in Gordon and Ramani's new model, which integrates the information processing approach to children's mathematical problem solving with the theory of embodied cognition, frequently used in gesture studies. While the model does not differentiate between speech and gesture input, it does differentiate between the gestures and speech that children produce: even with similar speech output, individual differences in math knowledge are proposed to affect children's gesture production.

Finally, the fact that findings about the benefit of gesture often conflict across studies is highlighted in the review article by Arachchige et al. The authors note methodological variations across the field and discuss how these differences may contribute to the heterogeneity of findings, limiting our ability to draw conclusions regarding underlying mechanisms.

Together, these articles demonstrate the critical role that gesture holds in human cognition and communication. Whether we are producing gestures ourselves, or observing those performed by others, gestures and speech interact in profound, and sometimes unexpected, ways. Gestures can aid comprehension and learning through semantic links with speech, and can have a similarly important role in the absence of speech. Gestures can affect social evaluations of speakers, but can sometimes come with associated cognitive costs. The effects of gesture must be examined in the context of individuals' cognitive characteristics, as well as differences in the gestures themselves. The articles in this collection further our understanding of human communication, highlighting the range of tasks, ages, individual differences and methods through which we may examine the integration of gesture and speech.

## Author Contributions

All authors listed have made a substantial, direct and intellectual contribution to the work, and approved it for publication.

## Conflict of Interest

The authors declare that the research was conducted in the absence of any commercial or financial relationships that could be construed as a potential conflict of interest.

## Publisher's Note

All claims expressed in this article are solely those of the authors and do not necessarily represent those of their affiliated organizations, or those of the publisher, the editors and the reviewers. Any product that may be evaluated in this article, or claim that may be made by its manufacturer, is not guaranteed or endorsed by the publisher.

## References

[B1] Cook S. W. Yip T. K. Goldin-Meadow S. (2010). Gesturing makes memories that last. J. Mem. Lang. 63, 465–475. 10.1016/j.jml.2010.07.002 21731176 PMC3124384

[B2] Goldin-Meadow S. Nusbaum H. Kelly S. D. Wagner S. (2001). Explaining math: gesturing lightens the load. Psychol. Sci. 12, 516–522. 10.1111/1467-9280.00395 11760141

[B3] Kelly S. D. (2001). Broadening the units of analysis in communication: speech and nonverbal behaviours in pragmatic comprehension. J. Child Lang. 28, 325–349. 10.1017/S0305000901004664 11449942

[B4] McNeill D. (1992). Hand and Mind: What Gestures Reveal About Thought. Chicago, IL: The University of Chicago Press.

